# Bis(2-hy­droxy­ethanaminium) tetra­chloridopalladate(II)

**DOI:** 10.1107/S1600536810045435

**Published:** 2010-11-13

**Authors:** Ilia A. Guzei, Lara C. Spencer, Margaret Yankey, James Darkwa

**Affiliations:** a Department of Chemistry, University of Wisconsin-Madison, 1101 University Ave, Madison, WI 53706, USA; b Department of Chemistry, University of Johannesburg, Auckland Park Kingsway Campus, Auckland Park 2006, South Africa

## Abstract

In the title compound, (C_2_H_8_NO)_2_[PdCl_4_], 2-hy­droxy­ethanaminium cations and tetra­chloridopalladate(II) dianions crystallize in a 2:1 ratio with the anion residing on a crystallographic inversion center. The cations and anions are linked in a complex three-dimensional framework by three types of strong hydrogen bonds (N—H⋯O, N—H⋯Cl, and O—H⋯Cl), which form various ring and chain patterns of up to the ternary graph-set level.

## Related literature

For the hydrolysis of imines in Schiff base first-row transition metal complexes, see: Chattopadhyay *et al.* (2007 ▶); Czaun *et al.* (2010 ▶); Guzei *et al.* (2010 ▶); Lee *et al.* (1948 ▶). For the use of Schiff base first-row transition metal complexes as amine protecting groups, see: Deng *et al.* (2002 ▶); Kurita (2001 ▶); Shelley *et al.* (1999 ▶). For geometrical parameter checks, see: Bruno *et al.* (2004 ▶). For *R* factor comparisons, see: Allen (2002 ▶). For graph-set notation, see: Bernstein *et al.* (1995 ▶).
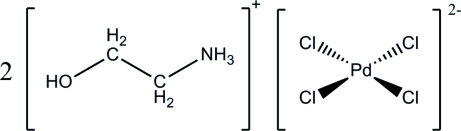


## Experimental

### 

#### Crystal data

(C_2_H_8_NO)_2_[PdCl_4_]
*M* *_r_* = 372.39
Monoclinic, 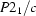

*a* = 8.9401 (4) Å
*b* = 8.1621 (4) Å
*c* = 8.5921 (4) Å
β = 103.445 (2)°
*V* = 609.78 (5) Å^3^
*Z* = 2
Mo *K*α radiation
μ = 2.37 mm^−1^
*T* = 100 K
0.30 × 0.10 × 0.06 mm

#### Data collection

Bruker SMART APEXII area-detector diffractometer
Absorption correction: analytical (*SADABS*; Bruker, 2001 ▶) *T*_min_ = 0.536, *T*_max_ = 0.871
14744 measured reflections
1851 independent reflections
1769 reflections with *I* > 2σ(*I*)
*R*_int_ = 0.024

#### Refinement

*R*[*F* ^2^ > 2σ(*F* ^2^)] = 0.012
*wR*(*F* ^2^) = 0.028
*S* = 0.98
1851 reflections
62 parameters
H-atom parameters constrained
Δρ_max_ = 0.43 e Å^−3^
Δρ_min_ = −0.50 e Å^−3^

### 

Data collection: *APEX2* (Bruker, 2007 ▶); cell refinement: *SAINT* (Bruker, 2007 ▶); data reduction: *SAINT*; program(s) used to solve structure: *SHELXTL* (Sheldrick, 2008 ▶); program(s) used to refine structure: *SHELXTL*, *OLEX2* (Dolomanov *et al.*, 2009 ▶) and *FCF_filter* (Guzei 2007 ▶); molecular graphics: *SHELXTL* and *DIAMOND* (Brandenburg, 2009 ▶); software used to prepare material for publication: *SHELXTL*, *publCIF* (Westrip, 2010 ▶) and *modiCIFer* (Guzei, 2007 ▶).

## Supplementary Material

Crystal structure: contains datablocks global, I. DOI: 10.1107/S1600536810045435/nk2068sup1.cif

Structure factors: contains datablocks I. DOI: 10.1107/S1600536810045435/nk2068Isup2.hkl

Additional supplementary materials:  crystallographic information; 3D view; checkCIF report

## Figures and Tables

**Table:** 

Pd1—Cl2	2.3074 (2)
Pd1—Cl1	2.3119 (3)

**Table:** 

Cl2^i^—Pd1—Cl1	89.409 (8)
Cl2—Pd1—Cl1	90.591 (9)

Symmetry code: (i) 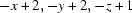.

**Table 2:** Hydrogen-bond geometry (Å, °)

*D*—H⋯*A*	*D*—H	H⋯*A*	*D*⋯*A*	*D*—H⋯*A*
N1—H1*A*⋯O1^ii^	0.91	1.97	2.8370 (12)	158
O1—H1⋯Cl1^iii^	0.84	2.35	3.1869 (8)	179
N1—H1*C*⋯Cl2	0.91	2.30	3.2048 (9)	170

Symmetry codes: (ii) 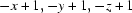; (iii) 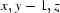.

**Fig. 1.**
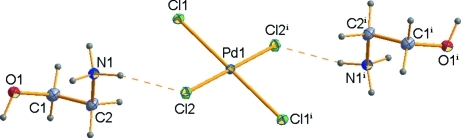
Molecular structure of (I). The thermal ellipsoids are shown at 50% probability level. Hydrogen bonds are shown with dashed lines. Symmetry transformations used to generate equivalent atoms: (i) -x + 2,-y + 2,-z + 1.

## supplementary crystallographic information

### Comment

Hydrolysis of imines in Schiff base first row transition metal complexes is now common (Chattopadhyay *et al.* 2007, Czaun *et al.*, 2010; Guzei *et al.*, 2010; Lee *et al.*, 1948) These metal complexes have been used to protect amines by first converting them to imines followed by metal assisted hydrolysis back to the amine (Deng *et al.*, 2002; Kurita, 2001; Shelley *et al.*, 1999). However, hydrolysis of imines by second row transition metal complexes is very rare. In an attempt to use 2,4-di-*tert*-butyl-6-{(2-hydroxyethylimino)methyl}phenol to prepare a palladium complex, we isolated the ammonium chloride salt of tetrachloropalladate, [C_2_H_8_NO]_2_^+^[PdCl_4_]^2-^, a result of the hydrolysis of the imine ligand.

The ionic title compound (I) consists of bis(2-hydroxyethanaminium) cations and tetrachloro-palladium(II) dianions in a 2:1 ratio. The tetrachloro-palladium(II) dianion resides on a crystallographic inversion center. The geometrical parameters of (I) are typical as confirmed by a *Mogul* geometrical check (Bruno *et al.*, 2004). Three types of hydrogen bonds, N1—H1A···O1,(*a*), N1—H1C···Cl2,(*b*), and O1—H1···Cl1,(*c*) form a three dimensional framework. The most easily visualized graph set motifs in the network include the primary ring pattern *R*^2^_2_(10) a->a->, three different secondary patterns formed by bonds b and c, the chain C^2^_2_(9) b^→^ c^←^, the chain C^4^_4_(18) b^→^c^←^c^←^ and the ring *R*^4^_4_(18) b^→^c^←^b^→^c^←^, and the ternary chain pattern C^3^_3_(8) a^→^c^→^b^←^  (Bernstein *et al.*, 1995).

The *R*-factor of the structural determination of (I) is a mere 1.18%. Data mining of the Cambridge Structural Database (Cambridge Structural Database, CSD, version 1.12, August 2010 update; Allen, 2002) found only 113 reported structural determinations with lower *R*-factors. This extremely low *R*-factor along with the unusually low standard uncertainties on the bond distances (fourth decimal place) and angles (third decimal place) are indicative of the high precision of this structure.

### Experimental

A solution of [PdCl_2_(NCMe)_2_] (0.11 g, 0.429 mmol) in dichloromethane (5 ml) was added to a solution of 2,4-di-*tert*-butyl-6-[(2-hydroxy-ethylimino)methyl]-phenol (0.12 g, 0.429 mmol) in dichloromethane (5 ml). The mixture was stirred at room temperature for 16 h, filtered, and the filtrate evaporated to dryness. Recrystallization of the residue from dichloromethane-hexane gave brown crystals over several days. Yield: 0.10 g (58%).

### Refinement

All H-atoms were placed in idealized locations with an O—H distance of 0.84 Å, N—H distances of 0.91 Å, and C—H distances of 0.99 Å. All H-atoms were refined as riding with appropriate thermal displacement coefficients *U*_iso_(H) = 1.5 times *U*_eq_(bearing atom) for the hydrogen atoms attached to oxygen atoms or 1.2 times *U*_eq_(bearing atom) for all hydrogen atoms attached to nitrogen or carbon atoms.

### Crystal data

**Table:**

(C_2_H_8_NO)_2_[PdCl_4_]	*F*(000) = 368
*M**_r_* = 372.39	*D*_x_ = 2.028 Mg m^−^^3^
Monoclinic, *P*2_1_/*c*	Mo *K*α radiation, λ = 0.71073 Å
Hall symbol: -P 2ybc	Cell parameters from 9951 reflections
*a* = 8.9401 (4) Å	θ = 3.4–30.6°
*b* = 8.1621 (4) Å	µ = 2.37 mm^−^^1^
*c* = 8.5921 (4) Å	*T* = 100 K
β = 103.445 (2)°	Block, orange
*V* = 609.78 (5) Å^3^	0.30 × 0.10 × 0.06 mm
*Z* = 2	

### Data collection

**Table:**

Bruker SMART APEXII area-detector diffractometer	1769 reflections with *I* > 2σ(*I*)
mirror optics	*R*_int_ = 0.024
0.60° ω and 0.6° φ scans	θ_max_ = 30.6°, θ_min_ = 3.4°
Absorption correction: analytical (*SADABS*; Bruker, 2001)	*h* = −12→12
*T*_min_ = 0.536, *T*_max_ = 0.871	*k* = −11→11
14744 measured reflections	*l* = −12→12
1851 independent reflections	

### Refinement

**Table:**

Refinement on *F*^2^	Primary atom site location: structure-invariant direct methods
Least-squares matrix: full	Secondary atom site location: difference Fourier map
*R*[*F*^2^ > 2σ(*F*^2^)] = 0.012	Hydrogen site location: inferred from neighbouring sites
*wR*(*F*^2^) = 0.028	H-atom parameters constrained
*S* = 0.98	*w* = 1/[σ^2^(*F*_o_^2^) + (0.0111*P*)^2^ + 0.3119*P*] where *P* = (*F*_o_^2^ + 2*F*_c_^2^)/3
1851 reflections	(Δ/σ)_max_ = 0.001
62 parameters	Δρ_max_ = 0.43 e Å^−^^3^
0 restraints	Δρ_min_ = −0.50 e Å^−^^3^

### Special details

**Table:**

Refinement. Refinement of *F*^2^ against ALL reflections. The weighted *R*-factor *wR* and goodness of fit *S* are based on *F*^2^, conventional *R*-factors *R* are based on *F*, with *F* set to zero for negative *F*^2^. The threshold expression of *F*^2^ > σ(*F*^2^) is used only for calculating *R*-factors(gt) *etc*. and is not relevant to the choice of reflections for refinement. *R*-factors based on *F*^2^ are statistically about twice as large as those based on *F*, and *R*- factors based on ALL data will be even larger.

### Fractional atomic coordinates and isotropic or equivalent isotropic displacement parameters (Å^2^)

**Table:**

	*x*	*y*	*z*	*U*_iso_*/*U*_eq_	
Pd1	1.0000	1.0000	0.5000	0.00979 (3)	
Cl1	0.73683 (3)	0.96899 (3)	0.46100 (3)	0.01355 (5)	
Cl2	1.04022 (3)	0.81392 (3)	0.70777 (3)	0.01331 (5)	
O1	0.57008 (9)	0.31533 (9)	0.44982 (9)	0.01677 (15)	
H1	0.6147	0.2245	0.4527	0.025*	
N1	0.74116 (10)	0.59194 (11)	0.58009 (10)	0.01328 (16)	
H1A	0.6491	0.6449	0.5570	0.016*	
H1C	0.8177	0.6650	0.6185	0.016*	
H1B	0.7404	0.5135	0.6552	0.016*	
C1	0.62509 (12)	0.42371 (14)	0.34444 (12)	0.01645 (19)	
H1E	0.5438	0.5041	0.2987	0.020*	
H1D	0.6487	0.3600	0.2551	0.020*	
C2	0.76792 (13)	0.51374 (13)	0.43181 (13)	0.01585 (19)	
H2A	0.8549	0.4359	0.4604	0.019*	
H2B	0.7951	0.5988	0.3610	0.019*	

### Atomic displacement parameters (Å^2^)

**Table:**

	*U*^11^	*U*^22^	*U*^33^	*U*^12^	*U*^13^	*U*^23^
Pd1	0.00874 (5)	0.00986 (5)	0.01049 (5)	0.00005 (3)	0.00164 (3)	−0.00051 (3)
Cl1	0.01008 (10)	0.01379 (10)	0.01650 (11)	0.00018 (8)	0.00250 (8)	0.00104 (8)
Cl2	0.01270 (10)	0.01301 (10)	0.01342 (10)	−0.00109 (8)	0.00143 (8)	0.00129 (7)
O1	0.0146 (3)	0.0126 (3)	0.0239 (4)	0.0011 (3)	0.0062 (3)	0.0002 (3)
N1	0.0124 (4)	0.0126 (4)	0.0146 (4)	−0.0007 (3)	0.0028 (3)	0.0006 (3)
C1	0.0148 (4)	0.0202 (5)	0.0141 (4)	0.0002 (4)	0.0029 (4)	−0.0009 (4)
C2	0.0136 (4)	0.0199 (5)	0.0153 (4)	−0.0011 (4)	0.0059 (4)	−0.0009 (4)

### Geometric parameters (Å, °)

**Table:**

Pd1—Cl2	2.3074 (2)	N1—H1B	0.9100
Pd1—Cl1	2.3119 (3)	C1—C2	1.5127 (15)
O1—C1	1.4322 (13)	C1—H1E	0.9900
O1—H1	0.8400	C1—H1D	0.9900
N1—C2	1.4932 (13)	C2—H2A	0.9900
N1—H1A	0.9100	C2—H2B	0.9900
N1—H1C	0.9100		
			
Cl2^i^—Pd1—Cl1	89.409 (8)	C2—C1—H1E	109.4
Cl2—Pd1—Cl1	90.591 (9)	O1—C1—H1D	109.4
C1—O1—H1	109.5	C2—C1—H1D	109.4
C2—N1—H1A	109.5	H1E—C1—H1D	108.0
C2—N1—H1C	109.5	N1—C2—C1	110.29 (8)
H1A—N1—H1C	109.5	N1—C2—H2A	109.6
C2—N1—H1B	109.5	C1—C2—H2A	109.6
H1A—N1—H1B	109.5	N1—C2—H2B	109.6
H1C—N1—H1B	109.5	C1—C2—H2B	109.6
O1—C1—C2	111.14 (8)	H2A—C2—H2B	108.1
O1—C1—H1E	109.4		
			
O1—C1—C2—N1	−51.28 (11)		

Symmetry codes: (i) −*x*+2, −*y*+2, −*z*+1.

### Hydrogen-bond geometry (Å, °)

**Table:**

*D*—H···*A*	*D*—H	H···*A*	*D*···*A*	*D*—H···*A*
N1—H1A···O1^ii^	0.91	1.97	2.8370 (12)	158
O1—H1···Cl1^iii^	0.84	2.35	3.1869 (8)	179
N1—H1C···Cl2	0.91	2.30	3.2048 (9)	170

Symmetry codes: (ii) −*x*+1, −*y*+1, −*z*+1; (iii) *x*, *y*−1, *z*.
